# Serial neuroimaging of brain growth and development in very preterm infants receiving tailored neuropromotive support in the NICU. Protocol for a prospective cohort study

**DOI:** 10.3389/fped.2023.1203579

**Published:** 2023-10-12

**Authors:** Carmina Erdei, Sara Cherkerzian, Roberta Pineda, Terrie E. Inder

**Affiliations:** ^1^ Department of Newborn Medicine, Brigham and Women’s Hospital, Boston, MA, United States; ^2^ Department of Pediatrics, Harvard Medical School, Boston, MA, United States; ^3^ Chan Division of Occupational Science and Occupational Therapy, University of Southern California, Los Angeles, CA, United States; ^4^ Division of Neonatology, Children’s Hospital of Orange County and University of California, Irvine, Irvine, CA, United States

**Keywords:** preterm, neurodevelopment, neurorehabilitation, multisensory experience, developmental care, NICU environment

## Abstract

**Introduction:**

Children born very preterm (VP) remain at risk for long-term neurodevelopmental impairment. Patterns of brain growth and injury, and how early neuropromotive therapies might mitigate developmental risk in VP infants remain insufficiently understood.

**Methods:**

This is a prospective cohort study of VP infants born at/before 32 weeks gestation. The study will enroll *n* = 75 consecutively-born VP infants in a level-III NICU. Exposed infants will be categorized into two groups (group 1: low-risk, *n* = 25 or group 2: high-risk, *n* = 25) based on the degree of neurological injury on early brain magnetic resonance imaging (MRI) at enrollment. Infants in the low-risk group (i.e., without significant injury defined as intraventricular hemorrhage with dilation, moderate or severe white matter injury, or cerebellar hemorrhage) will receive neurodevelopmental support utilizing the Supporting and Enhancing NICU Sensory Experiences (SENSE) program, while infants in the high-risk group (with neurological injury) will receive more intensive neurorehabilitative support (SENSE-plus). Age-specific, tailored sensory experiences will be facilitated contingently, preferentially by the infant's family with coaching from NICU staff. VP infants in exposure groups will undergo a brain MRI approximately every 2 weeks from enrollment until term-equivalent to monitor brain growth and evolution of injury. Exposed infants will be compared with a reference group (group 3: *n* = 25), i.e. VP infants whose families decline initial enrollment in SENSE, and subsequently undergo a term-equivalent brain MRI for other purposes. The primary aim of this study is characterization of term-equivalent brain growth and development among VP infants receiving NICU-based neuropromotive interventions compared to VP infants receiving the standard of care. Secondary aims include defining the timing and factors associated with total and regional brain growth on serial brain MRI among VP infants, (Aim 2), and using early imaging to tailor developmental intervention in the NICU while exploring associations with outcomes in VP infants at discharge and at two years corrected age (Aim 3).

**Discussion:**

This study will address gaps in understanding patterns of brain growth and injury drawing on serial MRI of hospitalized VP infants. These data will also explore the impact of intensive, tailored neuropromotive support delivered prior to term-equivalent on child and family outcomes.

## 1. Introduction

Preterm birth remains a public health emergency associated with high morbidity and mortality, leading to substantial emotional and financial burden for individuals, families, and communities (1–4). Infants born very preterm (VP, before 32 weeks gestation) often require several months of hospitalization in the neonatal intensive care unit (NICU). This is a time during which the preterm brain volume quadruples in size and is highly sensitive to both positive and negative environmental experiences (5). Yet, this is also a period that VP infants spend in the sensory-atypical environment of the NICU composed primarily of procedural touch, loud alarm noises, and bright lights (6). Alternatively, the NICU environment could consist of silence and a paucity of enriching stimuli such as human voice, touch, and interaction. Recent work suggests that adequate development of sensory functions in early infancy create the foundation for resilient higher-order cognitive, behavioral, and social-emotional processes later in life (7–9). Despite the attention that the neurosensory environment has received in the last decade, there is a paucity of safe and feasible early interventions supported by neurobiologically-based evidence to optimize the sensory environment and improve the neurodevelopmental trajectories of VP infants.

For VP infants in the NICU, optimal neural network development relies on appropriately timed, contingent, enriching sensory experiences which can decrease stress and optimize brain development during this important period (8). By term-equivalent age (TEA), VP infants have often experienced multiple morbidities following preterm birth, along with atypical neurosensory experiences, pain, and exposures to noxious substances which cumulatively impact brain growth and neurodevelopment (6, 10–12). Short-term consequences may include poorer neurological reflexes and quality of movement, hypertonia, or hypotonia (13), suboptimal orientation, state regulation, and social engagement (14), and impaired ability to manage stress (12, 15). Long-term, adverse neurodevelopmental outcomes of VP birth can range from more severe disabilities, including cerebral palsy; intellectual, language, or learning disability; hearing and vision impairments to high prevalence but low severity conditions such as developmental coordination disorders, fine motor deficits, and mild cognitive impairment (1–4, 16). Numerous factors including prenatal and perinatal events, neonatal morbidity, and postnatal exposures and sensory experiences are thought to be related to the neurobehavioral challenges experienced by VP children. However, there is a gap in understanding the specific neurobiological mechanisms that mediate these impairments.

Few prior studies have utilized longitudinal magnetic resonance imaging (MRI) during preterm infants’ NICU hospitalization in order to better understand the trajectories of structural growth and the development of brain injury. One recent study described the large increases in growth of cortical gray matter that were accompanied by decreases in relative unmyelinated white matter (17). Another study found that the most common neurological finding at TEA associated with preterm birth was diffuse white matter abnormality, although mechanisms and factors associated with these growth alterations and injuries remain to be elucidated (18). A limitation of both studies was the small number of MRI scans able to be performed for each infant, with many infants only undergoing one scan at the time of enrollment and one at TEA. One study investigated the structure–function relationship in preterm infants between MRI scans and clinical measures of motor, neurological and neurobehavioral outcomes (19). These investigators found associations between performance on standardized infant neurological assessments such as the General Movements Assessment (GMA), Hammersmith Neonatal Neurological Examination (HNNE), NICU Network Neurobehavioral Scale (NNNS), Premie-Neuro assessment, and Test of Infant Motor Performance (TIMP) and structural findings on both early and TEA brain MRI. While these previous studies using serial imaging techniques provide an important foundation for understanding individual brain development in the preterm infant, the lack of longitudinal MRI data over the entire course of NICU hospitalization highlights the need for further work to define the timing and factors associated with brain injury, as well as the pattern of early brain growth in the VP infant. Technological advances, including novel imaging analysis methods, are currently available which allow regional brain volumes to be investigated. In addition, previous studies relied on transporting small, fragile infants outside of the NICU environment to MRI scanners, which was a barrier to carrying out serial studies of VP infants. This barrier has now been overcome by novel within-NICU technologies and equipment available in some NICU settings (20).

Further, the relationship between multisensory experiences and the patterns of brain growth and injury experienced by VP infants before TEA remains insufficiently studied. The NICU design and operational model of care play a key role in regulating the timing, amount, type, and nature of multisensory experiences that preterm infants receive during NICU hospitalization, and these environmental factors integrate within the family-centered and family-integrated care frameworks (6). Evidence suggests that developmental care and therapies should ideally and preferentially be delivered by families with specialized staff guidance and support as indicated, in order to mitigate early risk, avoid harm, and optimize child and family outcomes (21–23). While direct family involvement is optimal for delivery of biologically-expected sensory experiences for VP infants, daily family presence in the NICU remains challenging, particularly in the United States (US) where parental family leave is often limited, and many families save such leave for after discharge from the NICU (24). In this context, NICU-based occupational therapists (OT), physical therapists (PT), speech and language pathologists (SLP), and other specialized NICU clinical staff play a critical role in the delivery of facilitated multisensory interventions for VP infants. This is achieved both directly through hands-on therapy as well as indirectly through family coaching when parents are present and engaged in the NICU (25).

Of note, a recent study indicated substantial variability in neonatal therapy staffing in NICUs across the US, calling for a need to benchmark developmental support in the NICU for more consistent service delivery (26). Further, as there is insufficient neurobiologically-based evidence to inform standardized developmental therapy protocols, significant practice variation remains. As such, an expert working group recently proposed a sensory-based intervention program for hospitalized preterm infants before TEA based on the available evidence and expert opinion consensus (27). Through this work, the Supporting and Enhancing NICU Sensory Experiences (SENSE) program (23, 27–29) was developed to engage parents in facilitating enriching, developmentally-appropriate sensory experiences with their preterm infants every day throughout their NICU hospitalization. Preliminary evidence indicates that protocolized implementation of this program for VP infants during their NICU hospitalization was associated with improved infant neurodevelopment and lower maternal stress at TEA (23) and better communication at one-year of age (28). While preliminary data on the implementation and impact of the SENSE program is promising, it has not yet been fully elucidated how multisensory experiences can be best integrated in NICU settings to support optimal brain growth and development in VP infants.

Therefore, the aims of this study are as follows:

1. To investigate brain growth and development among VP infants receiving a tailored neuropromotive support compared with those receiving the NICU standard of developmental care using a prospective cohort study design.
2. To define the timing and factors associated with total and regional brain growth on serial brain MRI among VP infants, along with key forms of brain injury prior to and at TEA. The patterns of brain injury monitored will include intraventricular hemorrhage, white matter abnormality, and cerebellar hemorrhage.
3. To utilize early brain imaging to categorize VP infants into low-risk or high-risk groups and tailor developmental support in the NICU according to their level of neurological risk, and subsequently explore associations between neuropromotive support and outcomes in VP infants and families at NICU discharge and at two years corrected age.

## 2. Methods and analysis

### 2.1. Informed consent

For each infant, we will plan to obtain written informed consent from one parent by a research study staff member. Consent documents will be available in English and Spanish. Infants not enrolled in the SENSE program (reference group) will consist of those who meet inclusion criteria, do not enroll in the exposure group, and who undergo a TEA MRI for another indication (clinical or other research). Enrollment of these reference subjects will occur prior to NICU discharge to enable collection of medical, sociodemographic, imaging data from the electronic medical record along with completion of outcome measures of the parents and infant at TEA. Infants in the reference group will receive the NICU standard of care throughout hospitalization. Infants in the SENSE program are intended to be enrolled by 32 weeks postmenstrual age (PMA), at which time a neuropromotive plan will be tailored based on the level on neurological injury noted on early imaging and implemented throughout the rest of the infant's hospitalization until TEA.

### 2.2. Design

This is a prospective cohort study of VP infants born at or before 32 weeks gestation in an academic, 66-bed level III NICU at Brigham and Women's Hospital in Boston, MA. The study will aim to enroll a total of 75 born consecutively infants divided in three study groups. Exposed infants will be categorized into two exposure groups (group 1: low-risk or group 2: high-risk) based on the degree of neurological injury on early brain magnetic resonance imaging (MRI) at enrollment, with 25 infants intended to enroll in each exposure group. Exposed infants in group 1 (low-risk, *n* = 25) will receive protocolized neurodevelopmental support utilizing the SENSE program. Exposed infants in group 2 (high-risk, *n* = 25) will receive the SENSE program in addition to enhanced neurorehabilitative support (SENSE-plus). The enhanced neurorehabilitation support will extend beyond the NICU standard of developmental care to include additional targeted visits that will consist of skilled neonatal developmental therapy sessions 4–5 times per week. The dose and frequency of sensory exposures in the groups receiving the SENSE program are anticipated to be delivered primarily by the infant's family, with education and support from NICU staff. Sensory experiences and neuropromotive interventions will also be tracked in both groups. Infants in the SENSE program will be compared with group 3 consisting of *n* = 25 reference (unexposed) infants whose families did not wish to enroll in the neuropromotive program, have undergone a term-equivalent brain MRI for clinical or other research purposes, and their parents subsequently provide informed consent to enrolling in the reference/unexposed group. Enrollment in each of the three groups will occur continuously until total sample size for each group is achieved.

### 2.3. Study subjects

The investigation team will recruit consecutive admissions of a total of 75 infants over the course of 2 years. Inclusion criteria consists of: VP infants born at or before 32 weeks of completed gestation based on the best obstetric estimate (or Ballard exam by clinical team if not available), with a birth weight between 0.5–4.5 kg, and deemed to be in stable condition per their clinical team. Families of all races or ethnicities will be included, with study materials available in English and Spanish. Infants will be excluded if they have a confirmed or suspected major congenital anomaly, a genetic syndrome, or congenital TORCH (Toxoplasmosis, Other Agents, Rubella, Cytomegalovirus, and Herpes Simplex) infection.

Study infants in the SENSE program (i.e., exposed group) will be included in one of two groups based on the level of neurological risk documented on early imaging (CUS or MRI):

1) Low-Risk group (*n* = 25) consisting of VP infants *without* significant neurological injury (defined as intraventricular hemorrhage (IVH) with any ventricular dilation, white matter injury (WMI) moderate or severe, cerebellar hemorrhage).
2) High-Risk group (*n* = 25) consisting of VP infants *with* significant neurological injury.

The reference (i.e., unexposed) group consists of *n* = 25 VP infants who receive the NICU standard of care who are eligible for approach, but do not consent to enrolling in the SENSE program and serial imaging portion of the study. These families will be reapproached for consent for a standard review medical records if their infant undergoes a brain MRI around TEA for an indication unrelated to the current study.

### 2.4. Study exposures

**Figure 1:**
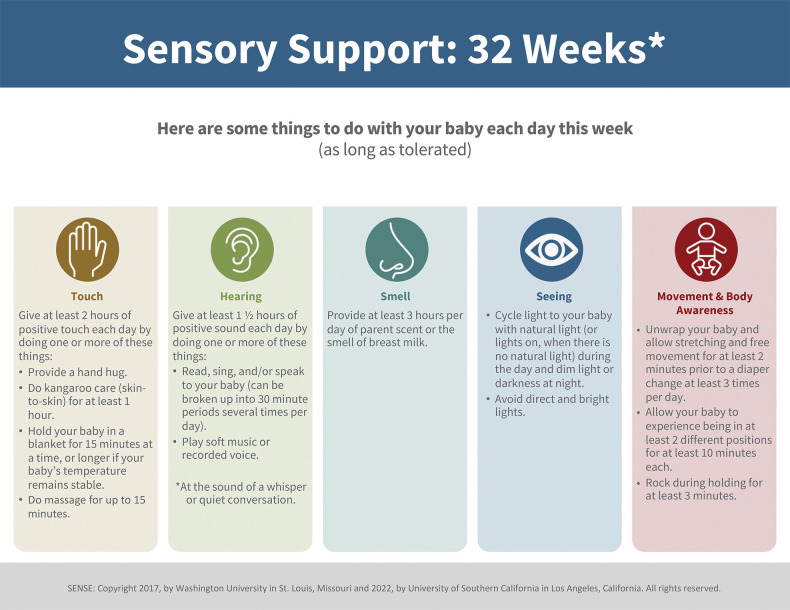
Sample SENSE plan (30): the week-by-week sensory exposure plan with tailored exposures as recommended for 32-week PMA.

A. SENSE program (low-risk group): this multisensory neurodevelopmental program incorporates expert recommended “doses” of tactile, auditory, olfactory, visual, vestibular, and kinesthetic exposures implemented every day while VP infants remain hospitalized in the NICU during a critical window of brain development before TEA. A sample of the weekly multisensory experience recommended plan is shown in Figure 1 (30). The SENSE program recommends contingent, appropriately timed, suggested amounts of each of the sensory experiences shown to be beneficial to the high-risk infant at each age and stage of development prior to TEA, drawing on existing research. These experiences include meaningful auditory exposures (e.g., parent or provider reading, talking, singing with infant), cycled lighting, skin-to-skin care and other human touch, gentle rocking, and opportunities for movement. This established neuropromotive program is intended to preferentially be conducted by parents, with support from trained clinical and research staff and NICU volunteers per unit standard of care when families are unable to be present daily and engaged in their infants’ care. Each experience is intended to be individually tailored to the infant's behavioral state, delivered contingently to the infant's cues. Experiences will be modified accordingly, or even discontinued if an infant is noted to show disengagement or stress cues, in keeping with supporting evidence for developmentally-sensitive practices for each week of PMA prior to TEA.
  The SENSE program will be facilitated for enrolled infants by a specialized team comprised of NICU neonatal therapists and trained research staff. The SENSE team will maintain a bedside log for tracking the multisensory experiences delivered each day and monitor progression towards achieving weekly goals. Family support will be incorporated to educate parents on the importance of meaningful experiences and coach parents on how to read and respond contingently to their infants’ behavioral cues during these interactions. Parents will also be coached and supported to optimize the time they are available to spend with their infant in the NICU, with a goal to deliver the majority of the recommended sensory experiences per the SENSE program whenever feasible and as tolerated by the infant. For families with limited ability to be present in the NICU, the neonatal therapy and research staff along with trained NICU cuddlers/volunteer staff will supplement the multisensory exposures to account for variances in parental involvement and optimize sensory exposures as/when indicated. The program also comprises a curated collection of parent educational materials on topics such as preterm infant developmental milestones, parenting in the NICU, and further in-depth guidance for families on how to tailor nurturing experiences contingent upon their infant's responses and tolerance.

B. SENSE-plus (high-risk group): this is conceptualized as an enhanced, intensive neurorehabilitation program for infants with early documented neurological injury. It includes all the elements of the SENSE program, with the addition of increased frequency of targeted motor (physical and occupational) therapy based on the type and location of brain injury on early MRI, as well as any differences or asymmetries noted on serial standardized neurological examination utilizing the Premie-Neuro assessment. This will differ from our unit's standard of care in which infants receive OT or PT 1–3 times per week until 34 weeks and 2–4 times per week thereafter, as the frequency and intensity will increase so that targeted motor therapy occurs 4–5 times per week. See Table 1 for a description of the support program and standardized neurological assessments for each study group.

**Table 1:** Developmental therapy, supportive services, and assessments by study group.

Reference Group	SENSE Program Groups	
	Low-Risk Group	High-Risk Group
Neonatal Therapy Standard Care	(Standard Care + “SENSE”)	(Standard Care + “SENSE-Plus”)
Developmentally sensitive bedside care OT or PT/Motor therapy: • 8–34 weeks PMA: total of 1–3 sessions per week • >34 weeks PMA: total of 2–4 sessions per week If known neurological injury or neurologic exam differences, or parent education and support need: consider increasing therapy to 2–4 times/week Neurologic assessments: • Brain MRI at TEA if <28 weeks GA at birth, or >28 weeks GA and additional risk factors per NICU guideline • TIMP at TEA by PT/OT in NICU • TIMP at 3–4 months PMA by OT in outpatient follow-up clinic SLP/Feeding therapy: • 33–34 weeks PMA: 1–2 times/week • >34 weeks PMA: 2–4 times/week Feeding assessment: • Assessment of feeding skills progression with FOIS-P (31) feeding scale	Neonatal therapy and assessment per reference Group, plus the following: • SENSE Program delivery with weekly targets for sensory experiences from enrollment to TEA • Therapist-guided parent training on reading infant's behavioral cues and contingent responses • Therapist-led education on positive auditory experiences (facilitating reading, music exposures per SENSE goals for GA) Neurologic assessments: • Brain MRI every 2 weeks from enrollment until TEA • Premie-Neuro every 2 weeks with MRI • TIMP and HNNE at TEA by PT/OT and research nurse • TIMP at 3–4 months PMA by OT in outpatient follow-up clinic Feeding assessment: • Assessment of feeding skills progression with FOIS-P (31) feeding scale	Neonatal therapy and assessment per Low-Risk Group, plus the following: Enhanced therapy involvement based on serial assessments and imaging results, as follows: • Additional 1–2 skilled OT or PT weekly sessions (totaling service delivery to 4–5 days/week) • Motor therapy goals targeted based on results of formal neurologic assessments, focus on areas requiring further therapist-guided interventions (e.g. postural control, midline orientation, symmetrical movements, motor experience) • OT/PT increased availability to facilitate day-shift routine care times with staff/parents (i.e. facilitation diaper changes, handling time) • Increased reading with infant several times a week (optimal: daily) • Enhanced family education with parent instructional videos

#### 2.4.1. Monitoring fidelity

Sensory experiences will be documented by families and staff using bedside log sheets to monitor fidelity and implementation of the intervention “doses” and frequency. The research study staff team will collect and review bedside logs regularly and provide feedback to staff and family regarding progress towards completion of weekly goals. The time-based duration of experiences received within each sensory category (tactile, auditory, visual, olfactory, and kinesthetic/vestibular) will be documented on bedside logs and quantified as a percentage of desired weekly goal. Further, therapy sessions will be tracked across groups to monitor frequency and duration as outlined in Table 1. These data will subsequently be transferred to a secure, HIPPA compliant Research Electronic Data Capture (REDCap) database for analyses and interpretation by the study staff.

#### 2.4.2. SENSE timeline

Infants in both the low- and high-risk groups will start receiving the SENSE or SENSE-plus program as early as possible following enrollment in the study (which is intended to occur by 32 weeks PMA). Based on early CUS and/or MRI results if available, the infant will receive the SENSE program if s/he qualifies for the low-risk group or the SENSE-plus if in the high-risk group. For each week from enrollment through TEA, updated multisensory support plans will be placed at the infant's bedside for the corresponding week of PMA (29, 30), which will subsequently be completed by families and staff.

### 2.5. Data collection and management

We will utilize the institution's Research Electronic Data Capture (REDCap), a secure, HIPAA compliant application, or a secure Mass General Brigham electronic research drive, for storage of all study data. Parental questionnaires will be directly administered and stored within the REDCap database. Brain MRI files will be transferred directly from the 1 Tesla or 3 Tesla scanner to a research secure server.

### 2.6. MRI protocol and procedures

The Aspect Embrace Neonatal MRI System is an FDA-approved device which uses innovative technology to safely and effectively perform infant brain imaging while they remain within the NICU. The BWH NICU houses a 1 Tesla in-unit MRI infant scanner with a built-in incubator, self-contained magnet, and continuous video monitoring.

#### 2.6.1. Timeline of MRI scans

**Figure 2:**
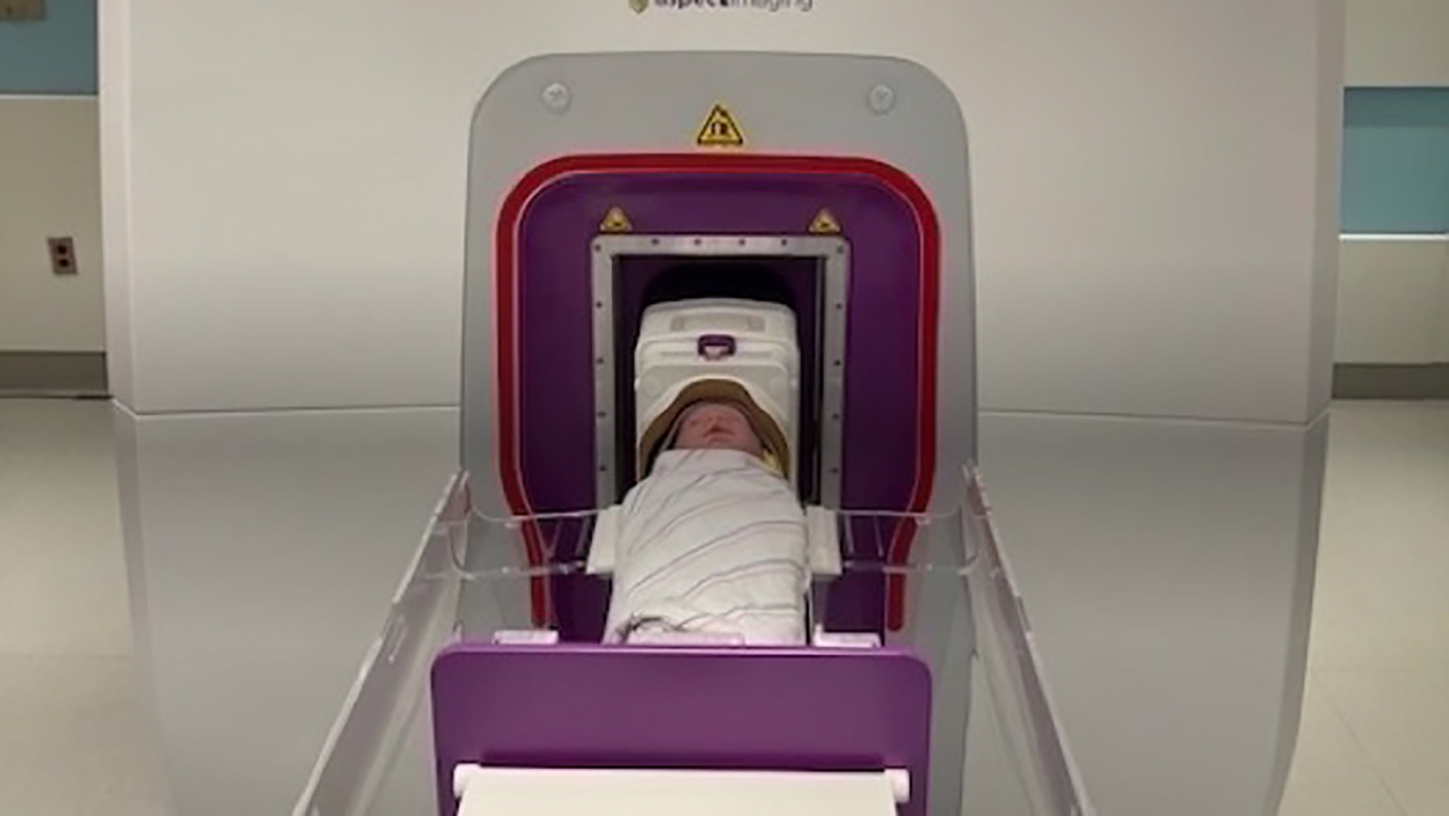
Procedure for brain MRI: infant wrapped in MRI-safe jacket is placed in Embrace Aspect scanner.

In our NICU, the clinical standard of care entails that babies born extremely preterm (before 28 weeks gestation), or VP (28–32 weeks gestation) with additional risk factors such as moderate-severe bronchopulmonary dysplasia, stage II or higher necrotizing enterocolitis, need for major surgery, severe growth restriction, or documented neurological abnormality on CUS among others, are considered for a TEA brain MRI. The TEA scan typically occurs between 38 and 42 weeks PMA or within the week of discharge, whichever comes first. Infants typically undergo brain MRI scanning without sedation in the study unit (20), using the documented “feed and wrap” method (32) (Figure 2). Serial CUS imaging is also obtained routinely in our unit for babies born before 32 weeks gestation per a clinical practice guideline, typically on days of life 1, 3, 7, 30, and 60. However, with only scarce evidence-based understanding of the etiology of many neuronal disorders available, findings on both serial CUS and TEA MRIs leave many unanswered questions in terms of etiology, timing, and evolution of injury prior to TEA, which have implications for the child's developmental trajectory and therapy needs post NICU discharge.

For both the low-risk and high-risk groups, we aim to obtain a first, early MRI once the infant is enrolled by 32 weeks PMA, and as soon as clinical status permits. Importantly, infants will need to be off mechanical ventilation and stable on continuous positive airway pressure as the maximal level of respiratory support to be able to undergo an in-unit MRI. This initial MRI will be used to understand the nature of any brain injury with systematic criteria for low or high neurodevelopmental risk as outlined above (i.e., enrolled VP infants with IVH with any ventricular dilation, moderate or severe WMI, and/or cerebellar hemorrhage will be assigned to the high-risk group; enrolled VP infants not meeting this criteria will be assigned to the low-risk group).

An infant will subsequently be assigned to the appropriate SENSE risk group (low-risk vs. high-risk) based on the results of the early CUS and MRI findings (if the first brain MRI is able to be completed at enrollment). If an infant is in a critical condition (i.e., receiving invasive mechanical ventilation or unstable on high level of noninvasive ventilation support) that does not permit safe scanning in the in-unit scanner, we will utilize the CUS results to determine assignment to an SENSE risk group, with plan for the first MRI imaging to be obtained as soon as the clinical status permits. If at any point an infant initially assigned to the low-risk group meets criteria for the high-risk classification based on identification of neurological injury on subsequent imaging, the infant will be transitioned to the SENSE-plus group to received enhanced neurorehabilitative support as outlined in Table 1. Enrolled VP infants in the low- or high-risk groups will be scanned at a frequency of approximately every 2 weeks, from the time of enrollment until TEA (approximately 38–42 weeks PMA) or until NICU discharge, to monitor brain growth and evolution of any injury. At TEA, infants enrolled in the low-risk or high-risk groups will undergo brain MRI in an off-unit Siemens Trio 3 Tesla scanner (Erlangen, Germany) equipped with a 16 channel pediatric head coil and 1 mm isotropic voxels. This schedule of serial imaging will inform characterization of patterns of brain growth of the VP infant throughout their NICU course, along with a broader understanding of the brain injury that occurs over the course of VP infants’ hospitalization.

### 2.7. Outcomes

A. NICU Phase

#### 2.7.1. MRIs

As above, we will aim for infants to undergo imaging every 2 weeks with at least 3 serial brain MRIs during the NICU phase: one at the time of enrollment in the study, and as soon as clinical condition allows; one or more interim scans at a frequency of approximately every 2 weeks; and a final scan at TEA in a 3 Tesla, off-unit scanner. We will use an automated segmentation technique (MANTiS) (33) to monitor the primary outcome i.e., evolution of total brain volumetrics as well as tissue-specific volumes of cortical grey matter, deep grey matter, white matter, hippocampus and cerebellum on sequential MRI scans of VP infants in the SENSE and reference groups. Secondary MRI outcomes will include fractional anisotropy in specific brain regions of interest which will be defined based on a validated method (34), and anticipated to include measures of white matter microstructure.

#### 2.7.2. Maternal outcomes

With parental consent, the study team will collect standard maternal demographic and pertinent perinatal course, along with infant clinical course data from the medical record. Demographic and family functioning measures will be collected from questionnaires given to parents at discharge. These will include: a general questionnaire regarding family composition and family-social risk factors, along with several standardized surveys to assess psychosocial and parenting experiences, including Hospital Anxiety and Depression Scale (39); Parental Stress Scale-NICU(40); and Parenting Sense of Competence Scale (41).

#### 2.7.3. Infant outcomes

As early neurobehavior is a good marker of outcomes at TEA (35), we will also obtain serial neurological assessments using the Premie-Neuro examination (36) around each MRI timepoint when possible. Around the time of the TEA brain MRI, an in-depth assessment of the infant's neurological status and neurobehavior will be conducted using the HNNE (37) and TIMP (38) standardized assessments. All neurological examinations will be performed by trained study staff and videorecorded for reliability and scoring purposes with parent consent. Examinations will be scored by an examiner blinded to infant group assignment.

B. Follow-up phase

As part of the standard care at BWH, children born VP are routinely offered interdisciplinary specialty care in the BWH NICU follow-up clinic for serial neurodevelopmental surveillance and family counseling during early childhood. For the purpose of this study, the consent form will include permission to review the medical record and document the results of these evaluations. If any subjects do not qualify for clinical follow-up, we will offer families a separate research-only follow-up visit. The developmental follow-up is intended to occur serially, up to around age 2 years corrected for prematurity, with a final evaluation window between 22 and 26 months corrected age. The primary neurodevelopmental outcome will be assessed using the Bayley Scales for Infant and Toddler Development, 4th Edition (42) standardized instrument, in conjunction with a neurological examination around 2 years corrected age. Parental questionnaire data will also be sought to reassess the family's psychosocial and parenting experiences, along with child's developmental skills with standardized instruments routinely used during the 2 year follow-up clinical assessments including the Ages and Stages Questionnaire-3: 24 month parent questionnaire (43), The Modified Checklist for Autism in Toddlers (44), and The Pediatric Eating Assessment Tool (45).

To promote retention and mitigate loss to follow-up, we will employ multiple strategies to enhance family compliance with visits and continued engagement. These include ongoing contact with participating families by study coordinators in the NICU and following discharge, mailing birthday cards, and small incentives for study assessment completion at NICU stage as described in the approved IRB protocol. For families who do not qualify for, or choose not to return for clinical follow-up, we will offer a one-time research-only visit at 2-year corrected age.

### 2.8. Sample size

For the primary aim, the proposed study has ≥80% power to detect differences in brain growth (total brain volume) on MRI at TEA between the SENSE (low- and high-risk groups combined) and reference groups of moderate to large effect sizes (Cohen's *d* = 0.70) using a two-tailed test with α=0.05. The power of the proposed study is sufficient to detect effect sizes reported in studies including a trial of early sensitivity training in parents of preterm infants (*n* = 45) which reported large effect sizes of *d* = 0.75–1.10 in white matter maturation and connectivity by ADC and FA between groups (46). The proposed study is further powered to detect small to moderate effect sizes (*d* = 0.41) between repeated brain measurements and moderate effect sizes (*d* = 0.59) in each SENSE subgroup individually (SENSE or SENSE-plus, *n* = 25 each) (Aim 2). Brain growth measured as total tissue, unmyelinated white matter, and cortical gray matter have been previously reported to exhibit moderate to very large effect sizes (*d* = 0.72–2.41) when comparing measures before and after 33–34 weeks gestational age (17).

### 2.9. Adverse events

Serious adverse events or study complications in this cohort study are not anticipated, as risks to subjects are minimal. Safety concerns will be addressed immediately with the principal investigator. The principal investigator will attend regular meetings with the study team where any safety issues are further discussed and then presented to the Institutional Review Board (IRB) if required. Severe or urgent safety concerns will be communicated immediately to the principal investigator who will report immediately to the IRB. Adverse events temporally related to participation in the study will be documented whether or not they are considered related to the test article.

### 2.10. Data analysis

Descriptive statistics of key demographic and clinical characteristics will be reported overall and by group status (exposed vs. reference) using means and standard deviations (or medians and interquartile ranges, as appropriate) for continuous and number and percentage for categorical variables. Differences by group status will be tested using chi square (or Fisher's exact test where cells <5) and Wilcoxon rank sum or Kruskal-Wallis tests, as appropriate, for categorical and continuous variables, respectively. Similar statistics will be reported by risk status among exposed infants including fidelity of implementation of the SENSE or SENSE-plus program in the low-risk and high-risk groups (>75%, 50%–75%, 25%–50%, and <25% compliance with total amount of recommended sensory exposure in each category). For the primary aim, outcome among infants in the SENSE program (exposed group) will be compared with the unexposed, similarly eligible infants whose families did not enroll in the neuropromotive program. Differences in outcome by group status will be analyzed using linear mixed models [or multinominal regression using generalized estimating equations (GEE) for ordinal categories of injury] to adjust for intrafamilial correlation among multiple births in models unadjusted and adjusted for potential bias (selection and confounding) by baseline covariates associated with both SENSE status and outcome. The study will also examine the difference between groups by exposure and high/low risk status, i.e., high risk: exposed vs. unexposed and low risk: exposed vs. unexposed. To address small sample size, we will utilize robust estimation methods. We will use a similar approach to assess differences between exposure groups for Aim 3 outcomes assessed at NICU discharge and at age two corrected age. Analysis of the change in repeated measures (Aim 2, SENSE groups only) will be modeled using linear mixed models and GEE, as appropriate, with fixed effects for SENSE group status, time, and the interaction between group and time. For assessment of change between two assessment time points, models will include fixed effects for group status and baseline assessment. We will use a similar approach to model Premie-Neuro exam scores (Aim 3) assessed at each serial MRI measurement for exposed infants. Analyses are exploratory and thus multiple comparisons will not be adjusted. Statistical analyses will be run using SAS v9.4 software (SAS Institute Inc., Cary, NC, USA) and IBM SPSS version 24.0 (IBM Corp, Armonk, New York, USA).

## 3. Discussion

To our knowledge, this is the first study to follow brain growth and injury in VP infants with serial MRI, and concurrently employ an evidence-based, tailored neuropromotive program before term age with the goal to optimize neurodevelopmental outcomes of VP infants. In this study, our aims are to investigate brain growth and development among VP infants receiving tailored neuropromotive support in the NICU compared to VP reference infants, and to characterize the timing and factors associated with total and regional brain growth and injury on serial brain MRI. Based on the results of initial imaging (initial brain MRI, or CUS if infant clinical status does not permit MRI imaging at the time of enrollment), infants will be assigned to either a low-risk or high-risk group, and subsequently receive protocolized, intensive in-NICU neuropromotive support tailored to their level of neurological risk until TEA. If subsequent imaging of an infant initially in the low-risk group indicates qualification for the high-risk group, the group assignment and the intensity of infant's neurodevelopmental support will be adjusted accordingly. The neuropromotive plan was designed based on an evidence-based program (28), adapted to include additional neurorehabilitative PT/OT support for high-risk infants with established neurological injury. We will explore the associations between risk-stratified NICU neuropromotive programming, and short-term (brain growth, neurobehavior, parent experience) and long-term (child development, family experience) outcomes of VP infants and their families.

The strengths of this study include the capability to obtain serial, in-unit MRI imaging, along with an expert-designed neuropromotive program that has been shown to impact clinical outcomes of VP infants in the NICU. Further, the program intensity will be amplified when higher grade neurological injury is identified, allowing for real-time intensive developmental therapy to facilitate neurorehabilitation. While some associations have been established between early interventions and neurodevelopmental outcomes, this study will contribute important mechanistic data which will broaden our understanding of how neuropromotive interventions can be best tailored for VP infants during a critical window of brain growth and development before TEA, for optimal downstream impact on child and family outcomes.

We acknowledge there are several limitations of this study. These include the prospective cohort study design which was chosen in order to achieve the primary aim of this study, i.e., to characterize brain growth and patterns of injury among VP infants. Infants in the current study are not randomized to SENSE intervention; rather, families of all eligible infants are approached for study consent. We recognize that infants whose families consent to the SENSE program (exposed) along with serial imaging may reasonably differ in their baseline characteristics from those who families do not initially consent to the SENSE program and serial imaging, but may subsequently agree to participate in the study as part of the unexposed, or reference group. We will address the potential for selection and confounding bias by evaluation of baseline demographic and clinical characteristics between group and adjusting statistical models as appropriate. Other limitations include the small size of the study with potentially limited power to detect a significant difference in effect between groups as well as the primary aim of this study being exploratory rather than hypothesis-driven; as such, observed associations between risk-stratified interventions and infant and/or family outcomes will be considered preliminary. Further, there may be imbalance in groups requiring oversampling in order to achieve at least 25 participants in each group.

## 4. Ethics and dissemination

The study protocol (#2019P003819) was approved through the IRB at Mass General

Brigham on 9/13/21. Dissemination of this work will occur through publication of short- (at NICU discharge) and long-term (2 years corrected age) infant and family outcomes in pediatric journals through a peer-review process. We anticipate that this work will contribute essential knowledge to further understand the relationships between brain development, brain plasticity, environmental experience, and outcomes in the vulnerable VP population. The results of this work may further inform optimal design of early neuropromotive interventions for hospitalized VP infants during a critical period of brain development prior to term age, with applicability in a large variety of NICU settings.

## Ethics statement

This study involving human subjects was approved by the Mass General Brigham Institutional Review Board. The study was conducted in accordance with the local legislation and institutional requirements. Written informed consent for participation in this study was provided by the participants' parents or legal guardians. Brigham and Women's Hospital Protocol Record 2019P003819, Serial Brain MRI in Hospitalized Preterm Infants, is registered in ClinicalTrials.gov Identifier: NCT06052865.
